# DIET AND COGNITION IN AGING: EFFECTS OF HIGH-FAT-SUGAR DIETS ON MEMORY AND EXECUTIVE FUNCTIONING

**DOI:** 10.1093/geroni/igae098.3042

**Published:** 2024-12-31

**Authors:** Selen Atak, Alyssa Boye, Anda Botoseneanu, Susana Pecina, Zhong-Xu Liu

**Affiliations:** University of Michigan Dearborn, Michigan, United States; University of Michigan Dearborn, Dearborn, Michigan, United States; University of Michigan-Dearborn, Dexter, Michigan, United States; University of Michigan Dearborn, Dearborn, Michigan, United States; University of Michigan Dearborn, Dearborn, Michigan, United States

## Abstract

High-fat and high-sugar (HFS) diets may affect the hippocampus, and consequently the hippocampus-dependent memory. Previously, we found that higher HFS diet consumption in young adults was associated with lower performance on hippocampus-dependent memory tasks, but not with their subjective memory report. However, whether similar associations exist in older adults has not been yet investigated. In this online study, 472 participants (age: 50 ~ 85 years; average = 62.6) reported their HFS consumption, measured by the Dietary Fat and Sugar Short Questionnaire, and their subjective memory issues. They also completed pattern-separation recognition and face-name associative memory tasks, which have been found to robustly engage the hippocampus. Participants also completed executive functioning tasks, i.e., Trail Making (TMT) and Stroop tasks. Results showed that different from young adults, higher HFS consumption was strongly associated with more subjective memory complaints in older adults after controlling for potential covariates. Moreover, age modulated associations between HFS consumption and memory/executive functioning. Specifically, higher HFS consumption was associated with worse hippocampus-dependent recognition memory and executive function, only in participants with age below 65, not in those with age above 65. Notably, HFS diet effects on recognition memory were also associated with participants’ health measured by multi-morbidity status, in that higher HFS predicted lower memory performance only in participants with no multi-morbidity. These results suggest that HFS diet effects on cognition are more complex in older than younger adults and call for further research to explore age-/health-related factors that uniquely modulate the effects of HFS diet in older adults.

